# Tumor Microenvironment in Diffuse Large B-Cell Lymphoma: Role and Prognosis

**DOI:** 10.1155/2019/8586354

**Published:** 2019-12-16

**Authors:** Alexandra Ioana Cioroianu, Patricia Irina Stinga, Liana Sticlaru, Mirela Daniela Cioplea, Luciana Nichita, Cristiana Popp, Florica Staniceanu

**Affiliations:** ^1^Department of Pathology, Colentina Clinical Hospital, 020125 Bucharest, Romania; ^2^Faculty of Dental Medicine, University of Medicine and Pharmacy “Carol Davila”, 020021 Bucharest, Romania

## Abstract

Diffuse large B-cell lymphoma (DLBCL) represents 30-40% of all non-Hodgkin lymphomas (NHL) and is a disease with an aggressive behavior. Because about one-third of DLBCL patients will be refractory or resistant to standard therapy, several studies focused on identification of new individual prognostic and risk stratification biomarkers and new potential therapeutic targets. In contrast to other types of cancers like carcinomas, where tumor microenvironment was widely investigated, its role in DLBCL pathogenesis and patient survival is still poorly understood, although few studies had promising results. The composition of TME and its interaction with neoplastic cells may explain the role of several genes (beta2-microglobulin gene, CD58 gene), receptor-like programmed cell death-1 (PD-1) and its ligand (PD-L1), or other cell components (Treg) in tumor evasion of immune surveillance, resulting in tumor progression. Also, it was found that “gene expression profile” of the microenvironmental cells, the phenotype of tumor-associated macrophages (TAM), the expression of matricellular proteins like SPARC and fibronectin, the overexpression of several types of matrix metalloproteinases (MMPs) like MMP-2 and MMP-9, or the tissue inhibitors of matrix metalloproteinases (TIMPs) may lead to a favorable or adverse outcome. With this review, we try to highlight the influence of microenvironment components over lymphoid clone progression and their prognostic impact in DLBCL patients.

## 1. Introduction

Diffuse large B-cell lymphoma (DLBCL) represents about 30-40% of non-Hodgkin lymphomas (NHL) [1]. Although DLBCL demonstrates an aggressive clinical course, using the established rituximab, cyclophosphamide, hydroxydaunorubicin, vincristine, and prednisone (R-CHOP) standard therapy, this neoplasm is curable in 60-70% of cases [1]. However, about one-third of these patients are refractory to this treatment. It is critical for them to find new therapeutic agents that alone or in addition to R-CHOP therapy may help to improve their survival or to provide an alternative for cases that are not eligible, are refractory, or have relapsed [2]. Recently, new molecular findings in DLBCL genetics have shown that these lymphomas comprise a group of disorders with specific signaling programs [1], and their first target was to identify new potential therapies with greater specificity and with lower toxicity [2].

Current research in this field is focused on identification of new individual prognostic and risk stratification biomarkers in order to predict the outcome and therapy response or that could indicate the patients who may be eligible for more aggressive therapies. Also, they may provide new perspective on current and future possible therapies.

Using gene expression profiling (GEP), Alizadeh et al. [3] found that DLBCL may be divided into two biologically and clinically molecular subgroups, with different prognoses and treatment responses. According to cell-of-origin (COO), these were defined as germinal center B-cell (GCB) (40-50%) or activated B-cell (ABC) (50-60%) subtypes [3]. Also, there was found a small unclassifiable group (10-15%) [3]. ABC DLBCL cases were found to have a poorer outcome than GCB DLBCL patients when treated with the standard therapy, with a 5-year survival of 44% for the ABC subtype and 87-92% for the GCB subtype [4, 5]. A recent discovery based on a new 20-gene assay permitted also the identification of the ABC vs. the GCB subgroup using formalin-fixed and paraffin-embedded tissue, a method which proved to be accurate and robust [6]. In addition, GCB DLBCLs were found to express genes of germinal center B cells, such as *CD10*, *LMO2*, or *BCL6*, and frequently, they were associated with *REL* amplification, *EZH2* mutation, or t(14;18) translocation [3, 7–13]. The pathogenesis of ABC DLBCLs was believed to be related to activation of the NF-*κ*B signaling pathway via the B-cell receptor (BCR) pathway, but recent studies demonstrated that NF-*κ*B may be expressed in both ABC and GCB DLBCL subgroups and is an adverse prognostic factor [7, 12–20]. *TNFAIP3*, *CARD11*, *CD79B*, *CD79A*, *TRAF2*, *MYD88*, and *REL* are the most commonly altered genes with an adverse impact in the ABC DLBCL subtype [7, 12, 13, 17–21].

Recently, several studies have focused on the potential role of the tumor microenvironment (TME) in DLBCL pathogenesis, but the results remained controversial. It is thought that the role of TME is based on the interactions between tumor cells and stromal elements (fibroblast, blood, and lymphatic vessels), extracellular matrixes, inflammatory, and immune cells (mast cells, macrophages, and T or B lymphocytes). The composition and spatial characteristics of the TME and the interaction between its components and lymphoma cells demonstrate significant heterogeneity depending on the type of lymphoma or the tissue or organ in which lymphoma arises and may have an important impact in the patient's survival, therapy response, and disease progression or relapse.

## 2. Immune Evasion

Immune evasion is a pathogenetic mechanism used by several types of cancers in their evolution, and avoidance of circulating T-lymphocytes (CTL) or the escape from NK cell recognition are the main processes implied. Challa-Malladi et al. [22] concluded that genetic alterations associated with lack of surface HLA-I and inactivation of the *beta2-microglobulin gene* (*B2M*) are present in 29% of DLBCL cases, leading to escape of tumor cells from CTL. *CD58*, the receptor of the natural killer (NK) cells or T cell CD2^+^, also has an important role in this process. 21% of DLBCLs, more frequently the ABC subtype, were found to have inactivation of the CD58 gene (*CD58*) that is implied in the loss of recognition of tumor cells by CTL and NK cells [22]. They concluded that both events may be coselected during lymphomagenesis and may be regarded as specific pathogenetic mechanisms [22].

*Programmed cell death-1* (*PD-1*) is a surface inhibitory receptor expressed by macrophages, dendritic cells, and T cells [23]. After PD-1 binds to PD-L1 (expressed on an antigen-presenting cell (APC) surface), it plays an important role in regulation of immune response by inhibiting cytokine production and cell-cycle progression in T cells [24].

Many studies tried to find the role of PDL-1 in the mechanism of immune evasion of aggressive B-cell lymphomas [25–28]. In DLBCL, PD-L1 expression was found in both tumor cells and microenvironmental cells, primarily macrophages, and had a controversial role [26, 27, 29, 30]. The DLBCL subgroup with PDL-1^+^ in tumor cells was associated with unfavorable prognostic factors like the non-GCB subtype, IPI high-risk group, elevated beta2-microglobulin, resistance to standard therapy, and reduced overall survival (OS) compared with the PD-1 negative subgroup [29–32] (see Table 1). On the other side, a favorable OS was seen in cases with PD-1 expression of a large number of tumor-infiltrating lymphocytes [32–34].

PD-L1^+^ tumor cells have other various mechanisms to escape T cell immune surveillance, the most important of them being the induction of apoptosis in some of the T cells through the PD-1/PD-L1 pathway [35]. Also, the expression of PD-L1 in myeloid dendritic cells (MDCs) induces T cell immune suppression in the tumor microenvironment [36]. Steidl et al. [25] have found that rearrangements of *CIITA* in B-cell lymphomas determine the overexpression of PD-1 and PD-L1 and may also lead to T cell immune avoidance.

*Regulatory T cells* (*Treg*) are other cellular components of TME that can contribute to the deprival of neoplastic cells from the effect of several proinflammatory stimuli released by nonneoplastic immune or inflammatory cells. One of the main functions of Tregs is the regulation of antitumor immune responses by inhibiting the cytokine production and suppressing the proliferation of CD8^+^ T cells, which may lead to an ineffective antitumor response and to the proliferation of cancer cells [37–39]. In DLBCL, the prognostic influence of FOXP3^+^ Treg is controversial, reported as being associated with a good prognosis in some studies or with an adverse outcome [40–42] or a trend toward a worse prognosis in other studies [43].

## 3. Stromal Gene Signature

Lenz et al. [4] identified a new gene expression profile of the nontumor cells, determining two different subgroups of DLBCL, predictive of survival and outcome in patients treated with R-CHOP [4].

High expression of “stromal-1 signature” was found in tumors with abundant extracellular matrix elements and a high number of macrophages. This subtype encodes elements of extracellular matrix, like osteonectin, various types of collagen and laminin, fibronectin, antiangiogenic factor thrombospondin, connective-tissue growth factor (CTGF), and remodeling proteins (MMP2, MMP9, MMP14, PLAU, and TIMP2) [4]. The “stromal-1” response was associated with a better prognosis [4].

“Stromal-2 signature” encodes markers of endothelial cells (CD31, von Willebrand factor), regulators of angiogenesis (vascular endothelial growth factor (VEGF) receptor, endothelial tyrosine kinase (TEK), and components of caveolae), and genes usually expressed in adipocytes, like *RBP4*, *ADIPOQ*, *PLIN*, and *FABP4* [4]. High expression of this subtype was correlated with poorer outcome and increased tumor blood vessel density [4].

Several studies tried to develop a new biologic prognostic model (BPM) and modified BPM (mBPM) based on COO and stromal-1 and stromal-2 responses, in order to determine DLBCL progression and response to therapy [44, 45]. The system uses three adverse prognostic markers—expression of SPARC, non-GCB subgroup, and high microvascular density [44, 45]. Cases with low score of BPM and mBPM showed a better survival rate [45] and higher rate of complete response to therapy [44] but without any impact on the patient's OS [44, 45].

Recently, Ciavarella et al. [46] analyzed GEP datasets from 175 cases of DLBCL using the computational method CIBERSORT to identify microenvironmental prognostic genes. Furthermore, they used the NanoString technology on FFPE to asses both TME genes and COO, in order to develop a reproducible assay [46]. They found that cases with higher proportions of myofibroblasts, dendritic cells (DCs), and CD4^+^ T cells had longer OS, independently of the COO [46]. In contrast, cases with a higher number of activated NK and plasma cells correlated with poorer outcome [46]. When they had integrated the two prognosticators, TME and COO, the survival prediction was improved [46].

## 4. Tumor-Associated Macrophages

The role of tumor-associated macrophages (TAMs) has been widely studied in the pathogenesis of various cancers, especially because of their controversial role. On the one side, they can kill tumor cells, but on the other side, they may favor tumor growth, invasion, and progression by inducing immunosuppression and synthesis of higher levels of angiogenic factors such as VEGF, interleukin 8 (IL-8), TNF-alfa, metalloproteases, and fibroblast growth factor 1 (FGF-1) [47].

In lymphomas, tumor cells release several soluble mediators, leading to continuous B-cell-receptor (BCR) stimulation and T cell and CD14^+^ monocyte recruitment and through them, to B-cell abnormal proliferation and rescue from apoptosis [48–51]. Khalifa et al. [52] found that lymphomas with an increased number of CD14^+^ monocytes and with loss of human leukocyte antigen-DR (HLA-DR) expression were more aggressive and more frequently associated with refractory disease or relapse to treatment.

There are also some discrepancies in the prognosis of TAM in DLBCL outcome, depending on the macrophage phenotype M1 (CD68/HLA-DR) or M2 (CD68/CD163). Riihijärvi et al. [53] found in their study that both CD68^+^ TAM and CD68 mRNA levels were associated with adverse prognostic factors for OS in patients treated with CHOP, but among patients that were treated with R-CHOP, the prognostic of CD68^+^ was favorable and the patients had improved OS. Marchesi et al. [54] (*n* = 61), Nam et al. [55] (*n* = 165), and Wada et al. [56] (*n* = 101) also concluded that M2 TAM is a significant factor for poor prognosis, being an independent predictor for shorter OS and PFS. On the other side, in several studies, no significant correlation was found between TAM and patient survival [57–59].

Marinaccio et al. [60] demonstrated opposing roles of inhibition and promotion of angiogenesis based on the M1 and M2 phenotypes of TAM, M1 macrophage having antitumor and antiangiogenic roles, and M2 macrophage acting as immunosuppressive and proangiogenic. Therefore, they concluded that since the expression of CD68 or CD163 is associated with an adverse outcome in patients treated with R-CHOP, double staining for CD68 and CD163 may be a better method of predicting outcomes of DLBCL [60].

*Legumain* is a cysteine protease, secreted by tumor cells undergoing hypoxia and also by TAM and is thought to have several roles in cancer pathogenesis. In tumors, the overexpression of legumain was found in correlation with angiogenesis, expansion of the tumor, and cleavage of the ECM [61, 62]. *In vitro* experiments of Shen et al. [62] showed first that M2 TAMs induced cleavage of ECM and formation of several vessel tubes demonstrating their proangiogenic role, but subsequent administration of legumain's inhibitors demonstrated that these effects were actually mediated by legumain.

## 5. Extracellular Matrix

Extracellular matrix (ECM) is composed by a mixture of several proteins, mineral deposits, and proteoglycans, synthesized by stromal cells, and has roles in supporting the cells and regulating intercellular interactions [49, 63, 64]. ECM composition is constantly changing by interactions between its components and different enzymes, contributing to progression of several types of malignancies [4, 49, 65]. Genes coding several ECM components, like collagens, laminin, metalloproteases, and matricellular proteins, were related to “stromal-1 signature” which have been associated with a favorable prognosis in DLBCL [4].

Among matricellular proteins, *SPARC* (secreted protein acidic rich in cysteine), also called osteonectin, is a marker expressed by a subset of macrophages and has ambiguous roles in tumor pathogenesis. It is considered in some studies as a tumor suppressor and in others a tumor promoter, favoring epithelial-to-mesenchymal transition (EMT), tissue invasion, or metastasis, depending on the tissue and cell type [66, 67].

In types of leukemia like myelogenous leukemia with MLL abnormalities that do not express SPARC, in pancreatic carcinoma, or in ovarian carcinoma, SPARC was associated with tumor suppression [68–70]. On the other side, lymphomas and types of leukemia with SPARC overexpression presented increased tumor growth [71]. In DLBCL, SPARC positivity of stromal cells was associated with longer OS and EFS than negative cases [4, 58]. Abdou et al. [44] reported high levels of SPARC in patients with adverse prognostic factors such as splenic involvement, but without any effect on patient overall survival, and concluded that DLBCL TEM could modulate tumor progression behavior.

In DLBCL, Brandt et al. [72] evaluated the expression of *fibronectin* (*Fn1*), another “stromal-1 gene,” and SPARC and concluded that combined immunohistochemical assessment of both of them is an important predictor of survival. They reported that “patients with double positive DLBCL had a significantly longer OS than the negative group and a better association with OS data than the expression of SPARC or Fn1 taken separately” [72].

The expression and synthesis of various types of *matrix metalloproteinases* (*MMPs*) in some aggressive B-cell lymphomas could be determined by neoplastic cell type and by the surrounding environment [72]. *Osteopontin* (*OPN*) is a TME matricellular protein, usually expressed by both normal and cancer cells that was demonstrated to have an important role in tumor invasion and metastasis through its function as a regulator of the enzymatic activity of MMPs [73–77]. Several studies suggested that overexpression of MMP-2 and MMP-9, upon their role in OPN-induced tumor invasion, is associated with an aggressive phenotype of cancers [78–81]. Their role in the dissemination and progression of aggressive NHL was also highlighted in the special literature [75, 82–84].

*IL-6* is another promoter of tumorigenesis [85–88], and in addition to OPN, it activates MMP-2 and MMP-9 and stimulates the expression of the tissue inhibitor of metalloproteinase (TIMP) by neoplastic and stromal cells [89]. IL-6 levels were reported to be associated with a poor prognosis in DLBCL [90]. *In vitro* studies conducted by Malaponte et al. [75] demonstrated that OPN, but not IL-6, stimulation was associated with increased MMP-9 and MMP-2 secretion and activation, suggesting that higher levels of IL-6 found in NHL and their role in proliferation, invasion, and migration of lymphoma cells may probably be attributed to the activation of other molecular pathways.

Usually, *tissue inhibitors of metalloproteinases* (*TIMPs*) have roles in maintaining the balance between ECM components through the inhibition of the MMP's activity, but several studies reported contradictory actions, especially in lymphomas, suggesting that they may actually contribute to tumor progression [49, 91, 92].

TIMP-1 is produced by both neoplastic lymphocytes and TME elements [49, 92, 93], and by activating different signaling pathways, it is an inhibitor of germinal center B-cell apoptosis and a promoter of neoplastic cell survival [92, 94]. The antiapoptotic role of TIMP-1, which may contribute to the poor prognosis of aggressive B-cell neoplasms, is determined by the binding of TIMP-1 to a putative cell-surface receptor, independent of its MMP inhibitory function [92].

TIMP-1 expression in DLBCL was assessed by Choi et al. [92], and they reported that it is an independent prognostic marker of poor prognosis and highlighted its possible role in the tumor progression but without any correlation with histogenetic origin or the presence of EBV infection.

## 6. Vasculogenesis

The angiogenic mechanism in DLBCL could be explained by different interactions of neoplastic cells and TME elements. The role of TAMs and mast cells in tumor progression and angiogenesis was demonstrated by their capacity of releasing several proangiogenic cytokines such as VEGF, IL-8, fibroblast growth factor 2 (FGF-2), and TNF-alfa [60, 95, 96]. Also, mast cells, by their role as regulators of MMPs and plasminogen activator (PA) activity, stimulate the proliferation of endothelial cells and the release of proangiogenic factors [60, 95–98].

Another angiogenic mechanism is related to a “stromal-2 signature” component—CXC chemokine ligand 12 (CXCL12) or stromal-cell-derived factor 1 (SDF-1)—a chemokine that can recruit CXCR4^+^ endothelial cells from the bone marrow [4, 99, 100]. The angiogenic role of the adipocyte-associated gene expression is related to adipocyte precursors that have the potential to differentiate into endothelial cells [101]. Finally, neoplastic B lymphocytes can also receive directly proliferation and/or survival signals through the overexpression of VEGF receptors [49, 102].

Upon these mechanisms, the relationship between MVD and DLBCL behavior was the object of many studies. High expression of the “stromal-2 gene signature” was found in cases with increased MVD, being correlated with an adverse outcome and with a shorter OS rate [4, 45, 60, 103]. In a cohort of 74 patients with DLBCL, Gomez-Gelvez et al. [43] reported contradictory results, high MVD being associated with better PFS and EFS. In the experiment conducted by Abdou et al. [44], MVD was found to be associated with poor prognostic parameters such as splenic involvement, high mitotic rate, and capsular invasion.

## 7. Conclusion

Our review of recent literature demonstrates once again that DLBCL is a disease with complex pathogenesis and behavior not only from the perspective of genetic alterations of lymphoid cells but also from the perspective of TME composition and its elements' interactions with neoplastic cells. These findings are important steps in understanding DLBCL pathogenesis and its unpredictable evolution. In addition to current COO classification and other prognostic markers, microenvironment assessment will discriminate better the subsets of patients with worse prognosis leading to the beginning of a new therapeutic era that will allow the administration of personalized therapy.

Patients with aggressive diseases that have relapsed or are refractory to current standard therapy may benefit from novel treatment strategies like antiangiogenic treatments, inhibition of legumain, administration of monoclonal antibodies targeting antigens of the myeloid-lineage cells, or immunotherapy targeting the PD-1/PD-L1 pathway.

Although the understanding of DLBCL biology has improved, the molecular mechanism by which several elements of TME confer aggressiveness is still poorly understood and further studies with larger cohorts and longer follow-up are recommended.

## Figures and Tables

**Table 1:** Characteristics of PDL-1^+^ and mPDL-1^+^ cases in various retrospective studies.

	Number of patients (*n*)	Treatment regimens	PDL-1^+^ tumor cells/mPDL-1^+^	OS/PFS	Specific results
Kiyasu et al. [29]	1253	R-CHOP/R-CHOP-like, CHOP	(i) *PDL-1^+^ tumor cells*: 10.5% (threshold ≥ 30%) (ii) *mPDL-1^+^*: 15.3% (threshold ≥ 20%)	(i) *OS HR*: 1.809 (CI: 1.051-3.112)	(i) *PDL-1^+^* is a poor prognostic factor; was significantly associated with the presence of B symptoms, IPI high-risk group, elevated serum soluble IL-2 receptor levels, EBV infection, and non-GCB subtype (ii) *mPDL-1^+^*: was found to be associated with IPI high-risk group, EBV infection, and non-GCB subtype

Hu et al. [30]	204	R-CHOP/R-CHOP-like, CHOP	(i) *PDL-1^+^ tumor cells*: 49% (threshold ≥ 5%) (ii) *mPDL-1^+^*: 21.6% (threshold ≥ 20%)	(i) *5-year OS* and *PFS*: 50% vs. 67.3% and 39.6% vs. 59.6%	(i) *PDL-1^+^* was found to be an independent risk factor for OS; it was associated with elevated beta2-microglobulin, resistance to first-line chemotherapy, and non-GCB subtype (ii) *mPDL-1^+^*: was associated with first-line chemotherapy resistance

Xing et al. [31]	86	R-CHOP (85%)	(i) *PDL-1^+^ tumor cells*: 16% (threshold ≥ 30%) (ii) *mPDL-1^+^*: 27% (threshold ≥ 5%)	(i) *Median OS and PFS*: 21 months and 18.5 months	(i) *PDL-1^+^* is a statistically significant factor for OS; it was also associated with higher initial staging, greater extralymphatic organ involvement and non-GCB subtype

Fang et al. [32]	76	R-CHOP/R-CHOP-like, surgery, and surgery^+^ chemotherapy	(i) *PDL-1^+^ tumor cells*: 26.3%	(i) *OS HR*: 2.547 (CI: 0.964-6.730)	(i) *PDL-1^+^* cases had a worse clinical outcome; it is not an independent prognostic marker for patients' OS
